# ABBaH teens: Activity Breaks for Brain Health in adolescents: study protocol for a randomized crossover trial

**DOI:** 10.1186/s13063-021-05972-5

**Published:** 2022-01-06

**Authors:** Emerald G. Heiland, Karin Kjellenberg, Olga Tarassova, Maria Fernström, Gisela Nyberg, Maria M. Ekblom, Björg Helgadottir, Örjan Ekblom

**Affiliations:** 1 Department of Physical Activity and Health, The Swedish School of Sport and Health Sciences (GIH), Lidingövägen 1, 11433 Stockholm, Sweden; 2 Department of Surgical Sciences, Medical Epidemiology, Uppsala University, Dag Hammarskjölds väg 14B, 75185 Uppsala, Sweden; 3 Department of Physiology, Nutrition, and Biomechanics, The Swedish School of Sport and Health Sciences (GIH), Lidingövägen 1, 11433 Stockholm, Sweden; 4 Department of Global Public Health, Karolinska Institutet, Solnavägen 1, 17177 Solna, Sweden; 5 Department of Neuroscience, Karolinska Institutet, Solnavägen 1, 17177 Solna, Sweden; 6 Division of Insurance Medicine, Department of Clinical Neuroscience, Karolinska Institutet, Solnavägen 1, 17177 Solna, Sweden

**Keywords:** Physical activity breaks, Sedentary, Cerebral blood flow, Cognitive function, fNIRS

## Abstract

**Background:**

Physical activity breaks are widely being implemented in school settings as a solution to increase academic performance and reduce sitting time. However, the underlying physiological mechanisms suggested to improve cognitive function from physical activity and the frequency, intensity, and duration of the breaks remain unknown. This study will investigate the effects of frequent, short physical activity breaks during prolonged sitting on task-related prefrontal cerebral blood flow, cognitive performance, and psychological factors. Additionally, the moderating and mediating effects of arterial stiffness on changes in cerebral blood flow will be tested.

**Methods:**

This is a protocol for a randomized crossover study that will recruit 16 adolescents (13–14 years old). Participants will undergo three different conditions in a randomized order, on three separate days, involving sitting 80 min with a different type of break every 17 min for 3 min. The breaks will consist of (1) seated social breaks, (2) simple resistance activities, and (3) step-up activities. Before and after the 80-min conditions, prefrontal cerebral blood flow changes will be measured using functional near-infrared spectroscopy (primary outcome), while performing working memory tasks (1-, 2-, and 3-back tests). Arterial stiffness (augmentation index and pulse wave velocity) and psychological factors will also be assessed pre and post the 80-min interventions.

**Discussion:**

Publication of this protocol will help to increase rigor in science. The results will inform regarding the underlying mechanisms driving the association between physical activity breaks and cognitive performance. This information can be used for designing effective and feasible interventions to be implemented in schools.

**Trial registration:**

www.ClinicalTrials.gov, NCT04552626. Retrospectively registered on September 21, 2020.

**Supplementary Information:**

The online version contains supplementary material available at 10.1186/s13063-021-05972-5.

## Background

Implementation of classroom-based physical activity breaks is being considered as a potential solution to combating sedentary behavior among adolescents and improving cognitive function and scholastic performance [1]. Focus on adolescents is pivotal as they are among the least studied groups in relation to physical activity and cognitive performance [2, 3]. Additionally, in Sweden, adolescents spend about 10.3 h per day sedentary, and only 43% of boys and 23% of girls meet the physical activity recommendations [4]. However, previous research on the effects of physical activity breaks on cognitive performance in adolescents has been inconclusive, mainly due to heterogeneity in study designs and methodology [1, 5–9].

Particular focus has been given to executive functions [10], as they are potential prerequisites for successful learning [11], emotional regulation [12], and child development [13]. Furthermore, among the cognitive functions, enhancement from physical activity has been most evident in the executive functions [14]. Working memory is a core component of the executive functions and involves holding information in mind and manipulating it to meet task goals [15], but has not been often studied in relation to physical activity nor in younger populations [2]. There is a clear connection between adolescents’ physical activity and cognitive ability [5], but knowledge is lacking on which intensity, frequency, and duration of physical activity is necessary to influence physiological mechanisms important for brain health and function.

One important mechanism that may explain the effects of physical activity on cognitive performance is changes in blood circulation in the cerebral cortex, especially in the frontal lobe (prefrontal), which is involved in solving cognitively demanding tasks [16, 17], but the majority of research has been in adults. Generally, results have revealed that a single bout of exercise can elicit immediate cognitive task-related increases on cerebral blood flow post exercise cessation [18–22], but also decreases or no effects have been observed [23, 24]. Reasons for differing results are partly related to the moderating effects of timing, duration, and intensity of the physical activity, and the timing and type of cognitive test performed.

Frequent, short physical activity breaks may be a more feasible alternative than a single bout, to counter the negative effects of prolonged sitting during a school day [25]. Studies have demonstrated positive effects of frequent, short physical activity breaks on peripheral vasculature in adults and older adults [26–30], but effects on cerebral vasculature remain elusive, especially in adolescents. In adults, studies performed using frequent, short physical activity breaks have been inconsistent, showing positive, negative, and no effects on cerebral blood flow [31–34] and on cognition [35–38]. Decreases in cerebral blood flow, with favorable effects on cognitive performance, have been seen in adult populations and suggest a potential neural efficiency adaptation [23, 24, 39, 40]. This suggests that after performing physical activity, efficiency in performing the cognitively demanding task increases so that less resources are required [39]. However, it remains unknown if a similar adaptation occurs in adolescents. Furthermore, studies that have measured cerebral blood flow have used indirect measurement modalities, such as transcranial Doppler ultrasound. One study using a more robust modality—functional near-infrared spectroscopy (fNIRS)—to study cerebral blood flow, found that male children with autism spectrum disorders had significant beneficial effects of a 20-min physical activity bout, compared to a sedentary counterpart, on prefrontal task-related cerebral blood flow [41]. Although performed in younger children (ages 8–10), another study found that after intermittent or continuous exercise, prefrontal-dependent cognitive performance improved compared to the baseline measure and this was significantly explained by alterations in cerebral blood flow assessed using fNIRS [42]. With fNIRS, it is possible to monitor cerebral blood flow by measuring changes in the attenuation of near-infrared light passing through the cortical tissue. Because fNIRS is non-invasive and does not require a certain posture or setting as in functional magnetic resonance imaging (fMRI), it is often used for brain imaging studies in infants and children [13]. It is considered a more feasible and robust option to measure cerebral blood flow changes, particularly when used in conjunction with short-separation channels, to eliminate the contaminating signal from blood flow in other tissues [43]. There are yet no studies investigating the effects of frequent, short physical activity breaks on hemodynamic changes using this technique in adolescents. We believe that the use of short-separation channels will produce partly different results.

Earlier studies have mainly focused on the effects of physical activity breaks only on cognitive performance, neglecting the underlying mechanisms [1]. Two studies have compared how different frequencies and durations of physical activity breaks affect cognitive performance. One study in children found that two 20-min breaks of moderate intensity were better than one or none for cognitive performance [44]. The other study found that 10–20 min long moderate-to-vigorous aerobic bouts were more beneficial for cognitive performance than that of a shorter duration (5 min) [8]. Favorable effects on working memory, as well as on well-being, were also observed in a school setting with a 15 min self-paced physical activity break at intensities near maximal exhaustion (the “Beep test”) [45]. Although, according to the inverted-*U* theory, high-intensity physical activity can produce unfavorable effects on cognitive performance [14]. Moderate-to-vigorous intensity is most favorable on prefrontal cognitive performance, especially when measured immediately after exercise cessation [14]. However, one study of young adults found that breaking up prolonged sitting with a 6-min bout of high-intensity interval exercise had no effect on a prefrontal-dependent cognitive test [46]. Studies incorporating both cerebral blood flow and cognitive performance are needed. A study in adults found that frequent, short walking breaks of moderate-to-vigorous intensity had beneficial effects on working memory after 3 h of sitting, although, with a coinciding decrease in cerebral blood flow [40]. However, it was not significantly different from the uninterrupted sitting condition. Nevertheless, significant improvements were seen in mood and alertness after the walking break condition compared with the uninterrupted sitting [40]. Thus, physical activity breaks may improve psychological health in settings where prolonged sitting occurs, but requires investigation in adolescents and whether favorable effects can also be demonstrated for working memory performance.

In addition, physical inactivity has been found to negatively affect vascular health (arterial stiffness) even among children [47, 48], whereas physical activity breaks may counter these effects [49, 50]. Arterial stiffness can also affect the cerebral vasculature and may therefore moderate or mediate effects of breaking up prolonged sitting on task-related changes in cerebral blood flow.

Furthermore, for activities to be sustainable in a real-world school setting, they need to be inclusive and non-demanding, but also possible to be performed on a regular basis. We therefore sought to investigate the effect of such activities. In turn, the results can reveal not only the potential underpinning mechanisms related to physical activity and working memory performance, but also provide insight into the type, intensity, and duration of physical activity breaks that can be implemented in schools to improve learning and psychological well-being throughout the school day.

### Aims and research questions

The main purpose of this study is to investigate how 80 min of prolonged sitting with and without frequent, short physical activity breaks of different intensities affects fNIRS-measured cerebral blood flow (measured as changes in oxygenated hemoglobin [oxy-Hb]) in the prefrontal cortex, in an ecologically valid mimicked classroom setting, during working memory tests of differential mental workloads in 13–14-year-olds.

Our primary research question is:

How does oxy-Hb in the prefrontal cortex during a working memory test change from before to after 80 min of prolonged sitting and after 80 min of sitting with physical activity breaks; and do these changes differ between these 80-min conditions?

Our secondary questions are:

1. Do physical activity breaks during prolonged sitting affect cognitive performance on the 1-, 2-, and 3-back tests; and self-reported psychological factors (stress, mood, alertness, and sleepiness)? Are these changes significantly different between conditions?
2. Are the changes in cognitive performance correlated with changes in oxy-Hb?
3. Does baseline arterial stiffness (measured as augmentation index and pulse wave velocity) moderate the change in cerebral blood flow and cognitive performance?
4. Does the change in arterial stiffness mediate the changes in cerebral blood flow and cognitive performance?

### Hypotheses

We hypothesize that:

- Task-related increases in oxy-Hb will occur after the conditions involving physical activity breaks and will either stay the same or decrease after the prolonged sitting, and these changes will be significantly greater for the higher intensity break.
- Cognitive performance, specifically reaction time and accuracy on the n-back tests, will improve after the physically active conditions, but decrease or remain the same after the prolonged sitting condition. The changes will significantly differ between sitting with or without breaks of different intensities, such that more favorable changes will occur when the breaks are of higher intensity.
- There will be a correlation between changes in oxy-Hb and cognitive performance.
- Psychological factors, including cortisol-measured stress, and self-reported mood, alertness, and sleepiness, will improve after the physical activity break conditions, which will be significantly different from the prolonged sitting condition, where there will be no change.
- Arterial stiffness will be reduced after the physically active break conditions, and these changes will be different from the prolonged sitting condition, where no changes will occur or an increase will be observed.
- Changes in oxy-Hb will be mediated by changes in arterial stiffness.
- Baseline arterial stiffness will moderate the change in oxy-Hb and cognitive performance, such that there will be a more pronounced change in those with lower arterial stiffness.

## Methods and design

### Trial design

A crossover randomized trial with three conditions will be carried out. All procedures will take place at the Swedish School of Sport and Health Sciences (GIH) in Stockholm, Sweden. Initially, each participant will attend a familiarization visit either at the laboratory at GIH with parental accompaniment or the researchers will visit the schools.

After the familiarization visit, participants will be assigned 3 experimental days for undergoing 3 different randomly ordered experimental conditions, each consisting of an 80-min sitting exposure. There will be a minimum washout period of 7 days between each visit [51, 52], to reduce carryover effects from the previous condition. Two participants will be tested simultaneously (with a 20-min gap between starting times) to increase efficiency and comfort for the participants. Participants will have a 24-h monitoring and standardization of their physical activity, sleep, and dinner prior to each experimental test day. Participants will receive compensation (600 SEK gift card) for their participation.

### Participants

Researchers will initially contact and visit interested schools in Stockholm and the greater Stockholm region, based on schools that have participated in a previous cross-sectional study at GIH, in order to minimize recruitment challenges. After agreement by teachers and the schools’ principals, consent forms will be sent home with the students to be signed by both parents prior to data collection. Students will then be invited to come to the laboratory at GIH with parental accompaniment for a familiarization session or researchers will visit the schools to introduce the study.

#### Eligibility criteria

Inclusion criteria are children aged 13 to 14 years. Those with ongoing medication that can affect the central or cerebrovascular circulation will be excluded, as well as those who have an ongoing infection, or are unable to apprehend information about the study or how to perform the tests. Students and parents must provide written informed consent prior to study commencement. Participants will be also informed that they may drop out from the study whenever they deem it necessary.

### Familiarization visit

Demographic and other data to describe the individual participant will be collected during the familiarization session including age, gender, height (not if familiarization was performed at the school) and weight without shoes, and a health screening questionnaire for potential contra-indicators. They will also be advised regarding what types of situations during testing that could lead to an immediate discontinuation to ensure safety in the laboratory (i.e., chest pain or similar; difficulties breathing; dizziness, confusion, slowed movements, feelings of sickness, coldness or cold sweaty skin; increase in heart rate not related to increased physical activity intensity; test leader decides to abort; physical or verbal signs of expressed exhaustion; or test equipment breaks). Blood pressure and heart rate will also be measured after 5 min of rest. In order to optimize fNIRS cap fitting, to increase reproducibility of fNIRS cap placement for each subsequent visit, and for head remodeling in future analyses, head circumference, nasion-to-inion, and left and right pre-auricular measurements will be taken, using a tape measure. The cognitive test will also be performed to increase familiarity and reduce practice effects.

A form with choices of food items, provided by Coop Sweden, will also be filled in (i.e., bread [soft or hard]; toppings [butter, cheese, red pepper, or cucumber]; yogurt or sour milk [filmjölk]; muesli or rye cereal; fruit [apple or clementine]), in order to standardize breakfast on experimental days. Additionally, participation in planned organized sport activities or other training will be noted to standardize physical activity the day before the experimental day. This information will also be used in order to determine which consistent weekday the participant would visit the lab, where they would have performed little or the same level of physical activity before each visit.

After the familiarization visit, allocation sequence for the order of experimental conditions will be assigned through individual randomization from a computer-generated random order, equally assigned to every participant. Randomization will be computer-generated by a researcher involved in the study and will be concealed through a password-protected document that will not be revealed until recruitment and the familiarization visit is completed. Researchers cannot be blinded in this type of study design or condition. Researchers within the study will have access to the allocation sequence list and will retrieve the randomization code prior to each experimental day in order to prepare the laboratory equipment.

### Pre-experimental day monitoring

In order to standardize the 24 h prior to each test day, participants will be advised to abstain from any heavy physical activity and will be asked to record details about their physical activity and sleep in a standardized diary. Participants will also wear activity monitors during the 24 h prior to test day to collect data on physical activity and sedentary behavior (hip-worn Actigraph GT3X+ and thigh-worn activPAL micro) and sleep (wrist-worn Actigraph GT3X+). Physical activity and sedentary variables from the Actigraph will include total physical activity and percentages of wear time spent in sedentary, light, and moderate-to-vigorous physical activity, according to standardized cut-points [53]. Physical activity variables from the activPAL micro monitor will include time spent sitting including in prolonged bouts (> 20 min), number of breaks from sitting each hour and time spent standing, stepping, and intense stepping. During night time, participants will wear the Actigraph GT3X+ on their left wrist [54]. Using the most common algorithm for sleep-wake scoring in children developed by Sadeh et al. [55], the following key aspects of sleep will be calculated: time to sleep onset, sleep efficiency, times waking up after sleep onset, and total sleep. Diaries will also be filled in to provide complementary details on placement time of activity monitors, sleep timing, and sleep behavior.

In order to standardize dietary intake and blood glucose levels, participants will be asked to record their dietary intake for the 24 h prior to each condition in a standardized food diary and closely match their dinner for each pre-condition day. They will also be asked to fast and abstain from caffeine after dinner the night before. A researcher will call or message the parents the day before the pre-condition day to remind about physical activity behavior the day before testing, taxi ride, standardizing the dinner, saliva samples, and health state of the participant (e.g., no COVID-19 symptoms). After each laboratory visit, a researcher will call or message to ensure that no influenza symptoms have developed in the household of the participant.

### Experimental conditions

To minimize physical activity on the morning of the visit, participants will be transported to the laboratory via taxi. On the morning of testing, participants will be asked to provide four saliva samples (for cortisol testing); immediately after waking up and at 30, 45, and 60 min thereafter. Once the participants arrive at the laboratory, a practice session of the cognitive tests will be performed, a saliva sample will be taken, and then a time-restricted, individually standardized breakfast (based on their food choices decided at the familiarization visit) will be provided. Participants at their initial visit will be advised to eat their preferred amount, which will be subsequently weighed and recorded. The portion sizes will be replicated at each visit. The activPAL activity monitor will be worn during the experimental conditions to monitor sitting time during the test day. Participants will be equipped with a chest strap heart rate monitor to be worn during the whole test day visit. After breakfast, pre-condition outcome measures will be collected.

**Fig. 1:**
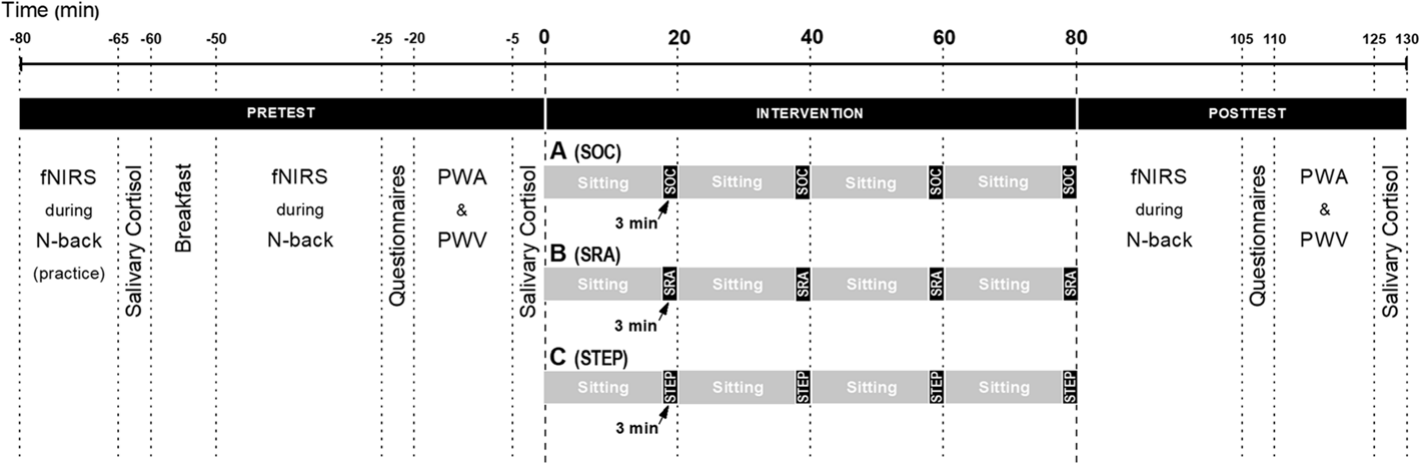
Experimental day procedures of the three conditions, including pretests, the 80-min intervention, and post-tests. FNIRS will be measured while participants perform working memory tests (*n*-back). Condition A: prolonged sitting with 3-min seated social breaks every 17 min (SOC); Condition B: prolonged sitting with 3-min simple resistance activities every 17 min (SRA); Condition C: prolonged sitting with 3-min step-up activities every 17 min (STEP). fNIRS, functional near-infrared spectroscopy; PWA, pulse wave analysis; PWV, pulse wave velocity

**Fig. 2:**
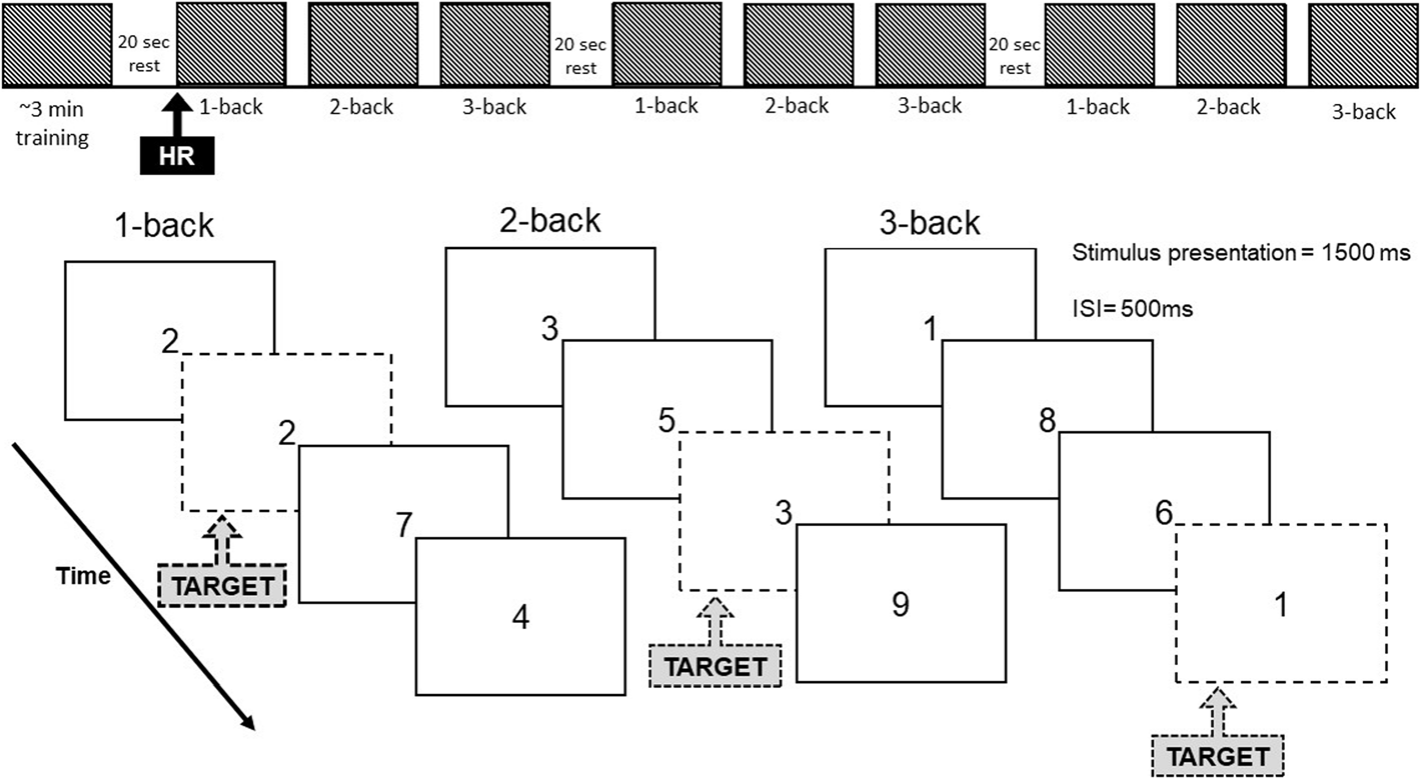
Schematic of working memory, *n*-back tests that will be administered while wearing the functional near-infrared spectroscopy cap to examine task-related changes in cerebral blood flow. The numerical n-back tests will precede with a short training session with feedback. Then 3 blocks of 1, 2, and 3-back tests (each block repeated 3 times), with a 20-s fixation screen between each of the 3 blocks and instructions before each n-back. Examples of correct responses for each *n*-back are labeled as “Target.” Heart rate (HR) will be measured at the start of the first 1-back. ISI, interstimuli interval

The three experimental conditions (summarized in Fig. 1) are (A) 80-min prolonged sitting with 3-min seated social breaks every 17 min (SOCIAL), (B) 80-min sitting with 3-min bouts of simple resistance activities every 17 min (SRA), or (C) 80-min sitting with 3-min bouts of moderate-intensity stepping every 17 min (STEP). During sitting, participants will be permitted to read a book, do schoolwork (from a laptop is permitted), and drink water, but not doze off or play games. The SOCIAL break (condition A) will consist of a 3-min chat between a research staff member and the participant while seated. For the SRA break (condition B), participants will be asked to complete 20-s body weight half-squats, 20-s calf raises, and 20-s gluteal contractions and knee raises repeated three times in sequential order by following standardized video instructions (as per [56]) with music. The STEP break (condition C) will be performed at moderate intensity at a pre-determined pace (around 110 beats per minute), using a standardized step (27.5 cm) following a standardized video with music in order to achieve a heart rate substantially higher than the SRA break. Toilet breaks and duration of the breaks will be recorded. Heart rate will be measured during the 80 min intervention at the 2:50 min time point during each break, and rate of perceived exertion (RPE) based on the Borg scale [57] will be evaluated immediately following each break. Heart rate will also be assessed once at the start of the first 1-back of the cognitive test (Fig. 2). In order to reduce the spread of COVID-19, certain hygienic and protective regulations and procedures will be followed. Details can be found in Additional file 1.

### Outcome measures

Outcome measures will be collected immediately prior to and immediately after each experimental condition.

#### Cerebral blood flow

The primary outcome measure will be the changes in oxygenated hemoglobin (oxy-Hb) using a non-invasive multi-channel continuous wave fNIRS instrument (portable NIRSport, 8-8 system, with short-separation channels, NIRx Medizintechnik GmbH, Berlin, Germany). The optodes will be positioned over the prefrontal cortex, with data sampled at 7.81 Hz at wavelengths 750 nm and 820 nm, for oxy-Hb and deoxygenated hemoglobin (deoxy-Hb), respectively. The fNIRS cap has 8 LED light sources and 8 detectors placed according to the standard 10–20 system, with a source-detector separation distance of 3 cm (Additional file 2). The eighth detector is split into 8 detectors for the additional short-separation optodes with a distance of 0.8 cm from each source (NIRx Medizintechnik GmbH, Berlin, Germany) to account for superficial blood flow [43, 58]. We chose to measure cerebral blood flow in the prefrontal cortex region as working memory tasks (such as the n-back) predominately activate this region [59–61]. After dimming the lights, and headphones placed on the participant to minimize auditory distractions, system calibration will be performed before each assessment using NIRStar 15.2 software, and using the predefined montage; and the fNIRS signals will be visually quality checked and noted during data collection for motion artifacts or lost channels. Cerebral blood flow measures will be taken simultaneously while the participant performs 3 cognitive tasks of different workloads (numerical 1-, 2-, and 3-back tests). Our primary outcome variables will be changes in oxy-Hb between the 1-back and 2-back, and between the 1-back and 3-back tests. We will also record deoxy-Hb concentrations. The frontal border of the fNIRS cap will be placed about 2 cm above the nasion point and centered on the Cz, in order to capture prefrontal cortex blood flow changes. We chose to examine oxy-Hb because this signal has been previously shown to correlate with blood flow better than the deoxygenated signal [62], and is more highly correlated with the blood-oxygen-level-dependent (BOLD) fMRI signal [62, 63], although both will be reported.

#### fNIRS calibration and data processing

Changes in cerebral blood flow (oxy-Hb) will be assessed using the MATLAB-based software NIRS Brain AnalyzIR toolbox (https://github.com/huppertt/nirs-toolbox) [64]. The data will be predicted on a block design, which is characterized by alternating periods of activity and rest to facilitate the acquisition of the fNIRS signals. Each trial will be preceded by 20 s of rest. Raw voltage will be converted to optical density, then to hemoglobin concentrations according to the modified Beer-Lambert law. After, the hemodynamic response function will be detected using an autoregressive model-based algorithm to solve for the general linear model (GLM), which allows for residuals of the GLM to be at random [65]. This approach uses pre-whitening filters using autoregressive models and employs iterative reweighted least squares, taking into account serial correlations, motion artifacts, and simultaneous regression of the short-separation channels. Gaussian functions will be used with a set standard deviation and separation of means according to the timeframe of the n-back task for the temporal basis of the function. First level statistics will be performed between each source-detector pair between the baseline (1-back) and the task conditions (2- and 3-back). False discovery rate (FDR) correction using a Benjamini-Hochberg procedure will be employed to correct for multiple comparisons at a critical level of < 0.05. Then, second-level group statistics will be performed on the regression coefficients obtained from the first-level statistics. Region-of-interest (ROI) statistics will be carried out to derive averages over all channels.

#### Cognitive performance

Cognitive performance in working memory will be assessed using a computerized *n*-back test [66], consisting of the 1-, 2-, and 3-back tests administered simultaneously with fNIRS measures. Participants will be required to indicate (via a key press within 2 s from stimulus onset) whether the digit presented on the screen was the same digit as the digit presented 1 stimulus previously (1-back), 2 stimuli previously (2-back), or 3 stimuli previously (3-back). Each digit will be presented for 1.5 s at an interstimuli interval of 500 ms. The n-back tests were created using E-Prime 2.0 (Psychology Software Tools). In order to reduce practice effects, participants will perform a shorter version (2 blocks) of the cognitive tests at the familiarization session and prior to data collection on each experimental day. The outcome variables for cognitive performance will include average reaction time (ms) and accuracy (average number of correct responses) for each test across 3 blocks of 20 digit sequences (see Fig. 2). There will be a 20-s rest between each set of 3 blocks, involving staring at a dot on the screen while internally counting up from 0 to allow for a short period of cognitive rest after instructions are presented on the screen. Practice tests with feedback will be provided before each testing followed by the actual testing without feedback. A task-based baseline was chosen (1-back), as rest is dynamic across age, and not considered an equalizer in younger age [67].

#### Arterial stiffness and pulse wave velocity

Arterial stiffness (defined as augmentation index [AIx]) and pulse wave velocity (PWV) will be measured using a SphygmoCor XCEL PWA/PWV system (AtCor Medical, Sydney, NSW, Australia) [68]. The technology is non-invasive, reproducible, and accurate using the radial pressure waveform to calculate the aortic pressure waveform. The AIx can be derived from the aortic pressure waveform and is defined as the difference between the first and the second systolic peak. PWV is considered the gold standard method for assessing arterial stiffness. PWV is calculated by the formula: PWV (m/s) = distance between measurement location (m)/transit time (s) [69]. First, after 2 min supine rest, blood pressure and AIx will be determined. Then, the distance from the suprasternal notch to the carotid site, from the suprasternal notch to the femoral site (top of the leg cuff), and from the femoral pulse to the top of the leg cuff will be measured using a measuring tape. These distances will be subsequently used to calculate PWV. Three high fidelity pressure waveforms will be recorded using a carotid tonometer with a leg cuff to capture blood pressure waveforms at the carotid and femoral sites. The average of the three high-quality recordings will be used to determine PWV (m/s).

### Other measures

*Stress* will be measured using salivary cortisol concentrations. Saliva samples (collected during the first hour after waking up on test days, prior to breakfast, and before and after the 80-min intervention) will be centrifuged for 10 min at 4 °C at 2800 rpm, and subsequently frozen at − 80 °C. Concentrations will be measured using the ELISA kit Abcam, ab154996. Questionnaires on mood, alertness, and sleepiness that have been previously used in studies with adolescents will be filled-in while seated after fNIRS measurement and prior to the 2-min supine rest before the arterial stiffness measurement. *Mood* will be assessed using the Positive and Negative Affect Scale (PANAS) [70–72], which includes 10 positive and 10 negative affects. Participants will rate on a 5-point scale (very slightly/not at all; a little; moderately; quite a bit; extremely) the extent to which they experience that mood at the present moment. The positive affect adjectives include interested, excited, strong, enthusiastic, proud, alert, inspired, determined, attentive, and active. The negative mood adjectives include distressed, upset, hostile, irritable, scared, jittery, afraid, ashamed, guilty, and nervous. *Alertness* will be measured using a simple 10-cm visual analog scale (VAS) [73], going from “not at all” to “completely alert”. *Sleepiness* will be measured using the Karolinska Sleepiness Questionnaire (KSS) [74–76], where participants will rate on a 9-point Likert scale their current level of sleepiness going from “extremely alert” to “very sleepy, great effort to keep awake, fighting sleep”.

### Data management

All participants will be assigned a unique identifier in order to remain anonymous. All personal data will be stored in encrypted files with access restricted to study staff. Original paper files will be stored in secure, locked cabinets on site. All data will be entered electronically at GIH. Data entered will be double-checked for errors at the time of entry, and changes made will be documented. Additional detection will be performed through the statistical software. Data ranges and consistency checks will be performed for data integrity. No specific auditing procedures are planned for this trial.

### Sample size

Studies with a similar design, and in children and youth are lacking. Thus, the sample size is based on studies of young and older adults. Using the data from these studies [18–20, 31, 77–79] software G*Power (Franz Faul, Universität Kiel, Germany, v 3.1.9.2) was used to calculate effect sizes. Effect sizes between 0.9 and 2.4 for the change in oxygenated hemoglobin were calculated, producing sample sizes between 6 and 13 participants (given an *α* = 0.05 and *β* = 0.8 assuming a two-tailed test). However, in order to have high enough variance within this age group, we will recruit 16 participants.

### Statistical methods

Mixed effects models with subject as a random effect to assess within condition differences will be performed, and to examine time-by-condition interaction effects. Changes in arterial stiffness and PWV will be assessed in relation to cerebral blood flow changes in the mixed models.

Exploratory analyses will be performed excluding the short-separation regressors and ROI averages to compare to the main analyses.

Secondary outcomes (i.e., cognitive function, psychological factors, stress), will be assessed using mixed models, with the subject as a random effect, looking at both the within condition changes and the time-by-condition interaction effects. Correlational analyses will be performed to investigate the association between changes in cognitive function and cerebral blood flow if significant changes are seen. Maximum likelihood estimation will be used in the mixed models to handle missing data at random. Subsequent sensitivity analyses will be performed if necessary.

## Discussion

As interventions to improve school-based performance have become a priority, greater understanding is needed on the effects of physical activity breaks on cognitive performance and the underlying mechanisms. This study will provide insight into the effects of frequent, short physical activity breaks during a prolonged period of sitting on cerebral blood flow. This feasible approach to physical activity breaks during the day may be promising for implementation in schools where children spend most of their time and often in a seated posture. With emerging evidence regarding increasing sedentary behavior and poor academic performance, strategies to break up sitting are needed that are not cumbersome. Furthermore, understanding the underlying mechanisms that may be driving the association can be pivotal so that more informed interventions can be developed and precise recommendations supplied to benefit schoolchildren's health and psychological well-being and reduce sedentary behavior. The results from this study will also tackle some of the lingering questions regarding other intensities, durations, and frequencies of physical activity breaks. Moreover, the effects on psychological well-being will also be investigated, which are important in order to assess adolescents’ subjective well-being. Assessing whether differences in arterial stiffness, even at a young age, mediates or moderates cerebral blood flow may add understanding into the mechanisms underpinning effects of prolonged sitting on cerebral blood flow, mood, and working memory performance.

There are many strengths of this trial, such as the use of state-of-the-art technology in measuring cerebral blood flow—fNIRS with short-separation channels. This can give more accurate results that have been lacking in previous studies [31–34, 41, 80]. The mimicking of the school environment (duration of 2 school lessons), and use of devices to measure physical activity and sedentary behavior provide more robust and ecologically valid estimates of pre-day physical activity and sleep behaviors. Additionally, the exercise paradigms are readily available to be implemented in school settings. Although the inclusion of a control group might be considered in a more potent study design, the randomized crossover design is a more attractive alternative as it allows the participants to be their own controls, thus reducing variability between individuals. Mind wandering may affect the participants as well as potential anticipatory affects due to awareness of the condition and subsequent conditions. However, the use of counting during the rest periods and randomization of the order of the conditions will help in minimizing these contaminations. Repositioning of the cap during test days may also reduce accuracy in measurement from pre-test to post-test, however, the use of a marker on the forehead will help to reduce misalignment. In addition, only the prefrontal cortex will be measured in this study, thus cortical activity in other regions cannot be measured. The experiments are to mimic a school day; therefore, generalizability to other settings or days of the week (i.e., weekend) needs to be carefully considered. Finally, this is the first study of this kind therefore subsequent studies will be required to confirm the results.

## Trial status

This is the first version of the protocol. The trial was last amended in the registry December 16, 2020, on https://clinicaltrials.gov/ct2/show/NCT04552626?term=ABBaH&draw=2&rank=1. Recruitment began on September 15, 2020, and was completed in January 2021. The COVID-19 situation prompted us to urgently begin with recruitment and data collection, thus placing protocol submission alongside said tasks and effectively delayed with regard to our preferred schedule.

## Supplementary Information

**Additional file 1.** Laboratory procedures and regulations concerning COVID-19 (.pdf)

**Additional file 2.** Prefrontal cortex montage setup for the fNIRS cap (.pdf)

**Additional file 3.** SPIRIT Checklist (.docx)

## Data Availability

Upon reasonable request, data may be made available by contacting the project investigator ÖE.

## Abbreviations

CBF: Cerebral blood flow
fNIRS: Functional near-infrared spectroscopy
fMRI: Functional magnetic resonance imaging
oxy-Hb: Oxygenated hemoglobin
deoxy-Hb: Deoxygenated hemoglobin
GLM: General linear model
BOLD: Blood-oxygen-level-dependent
ROI: Region-of-interest
AIx: Augmentation index
PWV: Pulse wave velocity
PANAS: Positive and Negative Affect Scale
VAS: Visual analog scale
KSS: Karolinska Sleepiness Scale
