# Supplementary material for: ABBaH teens: Activity Breaks for Brain Health in adolescents: study protocol for a randomized crossover trial
Source: Trials. 2022 Jan 6;23:22. doi: 10.1186/s13063-021-05972-5 (PMC8733916; doi:10.1186/s13063-021-05972-5)
Supplement: Supplementary file 1 — Additional file 1. Laboratory procedures and regulations concerning COVID-19 (.pdf) [file 13063_2021_5972_MOESM1_ESM.pdf]

Additional file 1

Laboratory procedures and regulations concerning COVID-19

Prior to each laboratory visit, participants will be contacted to determine if they are well and have no symptoms related to COVID-19. On each test day, before participants arrive at the laboratory all unnecessary equipment will be removed from the test area and all surfaces in the test areas will be cleaned and disinfected. Data collectors will wash and disinfect their hands, and put on protective clothing according to regulations set in place by the Swedish School of Sport and Health Sciences (GIH) before participants arrive. These regulations involve that data collectors wear masks, gloves, robes, and visors if in close contact with the participants, otherwise if only near the participant (1-2 meters) a mask and gloves are required. Upon arrival at the laboratory, participants will be required to disinfect their hands. The laboratory will be divided into different areas in order to increase efficiency in flow with minimal contact between persons, and with an assigned area for changing/disposing of protective clothing, and hand washing/disinfecting. After each visit, participants will be required to disinfect their hands before leaving the laboratory. Data collectors will clean and disinfect all test areas, and garbage will be removed immediately. A checklist for each participant's visit will be filled out and signed by all data collectors and the assigned test leader during and after each visit to confirm that regulations and procedures were followed. Participants will be informed that they must contact the test leader if any COVID-19 related symptoms arise within 7 days of visiting the laboratory. In addition, participants will be contacted within 7 days of visiting the laboratory to confirm that the participants are without symptoms related to COVID-19. If symptoms become apparent, the responsible person for the laboratory and the research group leader will be informed.
