# Supplementary material for: ABBaH teens: Activity Breaks for Brain Health in adolescents: study protocol for a randomized crossover trial
Source: Trials. 2022 Jan 6;23:22. doi: 10.1186/s13063-021-05972-5 (PMC8733916; doi:10.1186/s13063-021-05972-5)

Additional file 2

Prefrontal cortex montage setup for the fNIRS cap with 8 LED light sources (S1-S8) and 7 detectors (D1-D7) placed according to the standard 10-20 system, with a source-detector separation of 3 cm. An eighth detector was split into eight short-separation detectors (SD1-SD8) placed at a distance of 0.8 cm from each source, to capture superficial blood flow.

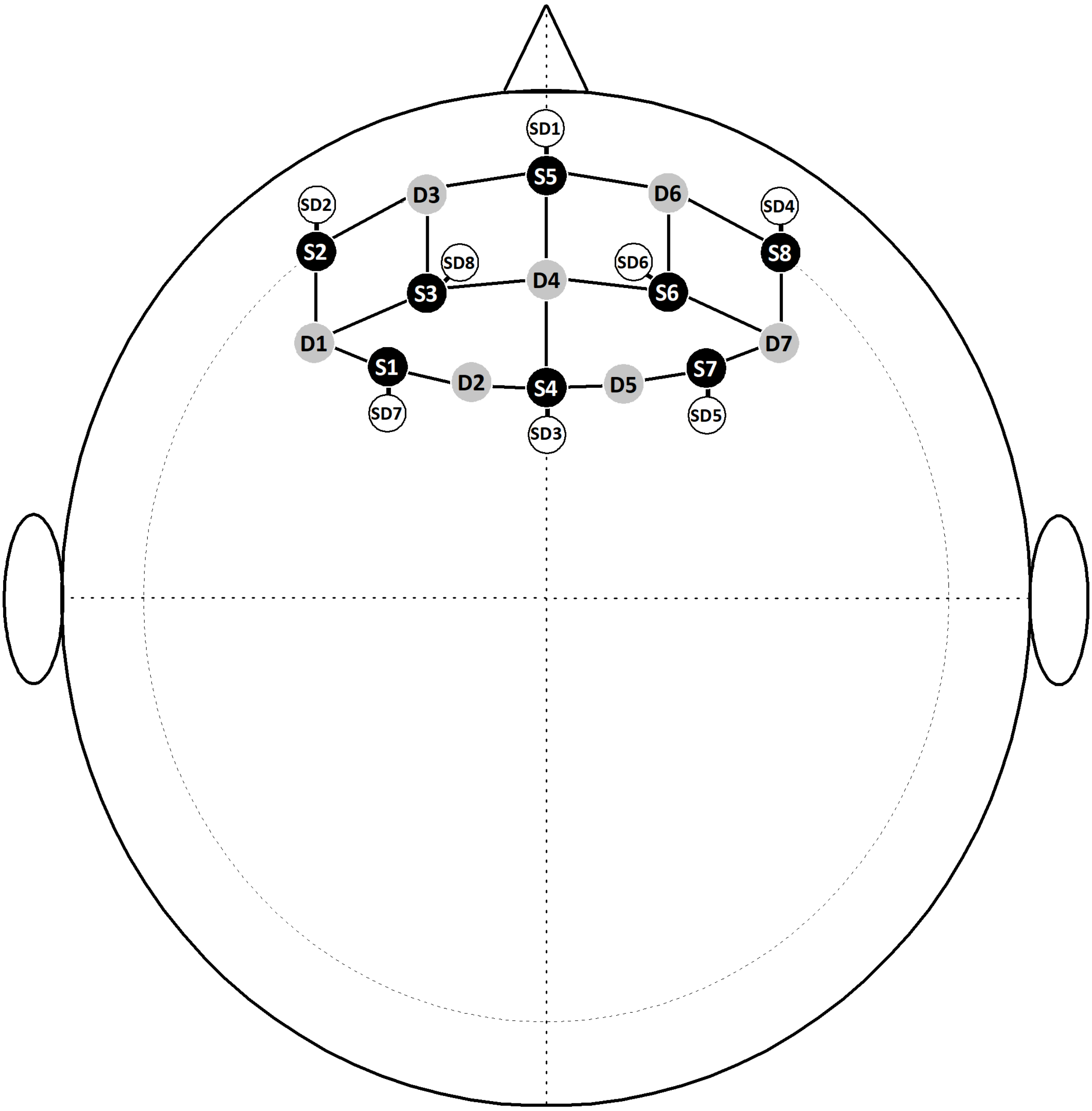

Supplement: Supplementary file 2 — Additional file 2. Prefrontal cortex montage setup for the fNIRS cap (.pdf) [file 13063_2021_5972_MOESM2_ESM.pdf]
